# PPAR****γ**** as a Potential Target to Treat Airway Mucus Hypersecretion in Chronic Airway Inflammatory Diseases

**DOI:** 10.1155/2012/256874

**Published:** 2012-06-17

**Authors:** Yongchun Shen, Lei Chen, Tao Wang, Fuqiang Wen

**Affiliations:** Division of Pulmonary Diseases, State Key Laboratory of Biotherapy of China and Department of Respiratory Medicine, West China Hospital, Sichuan University, Chengdu 610041, China

## Abstract

Airway mucus hypersecretion (AMH) is a key pathophysiological feature of chronic airway inflammatory diseases such as bronchial asthma, cystic fibrosis, and chronic obstructive pulmonary disease. AMH contributes to the pathogenesis of chronic airway inflammatory diseases, and it is associated with reduced lung function and high rates of hospitalization and mortality. It has been suggested that AMH should be a target in the treatment of chronic airway inflammatory diseases. Recent evidence suggests that a key regulator of airway inflammation, hyperresponsiveness, and remodeling is peroxisome proliferator-activated receptor gamma (PPAR**γ**), a ligand-activated transcription factor that regulates adipocyte differentiation and lipid metabolism. PPAR**γ** is expressed in structural, immune, and inflammatory cells in the lung. PPAR**γ** is involved in mucin production, and PPAR**γ** agonists can inhibit mucin synthesis both *in vitro* and *in vivo*. These findings suggest that PPAR**γ** is a novel target in the treatment of AMH and that further work on this transcription factor may lead to new therapies for chronic airway inflammatory diseases.

## 1. Introduction 

Airway mucus hypersecretion (AMH) is a common pathological feature in chronic airway inflammatory diseases such as asthma, chronic obstructive pulmonary disease (COPD), and cystic fibrosis (CF). Growing studies have suggested that AMH is associated with the progression of chronic airway inflammatory diseases and it is a significant contributor to morbidity and mortality. To control, AMH plays an important role in the treatment of chronic airway inflammatory diseases; however, effective therapies that target AMH are lacking [1, 2].

Peroxisome proliferator-activated receptors (PPARs) are a family of ligand-activated transcription factors belonging to the nuclear hormone receptor family. PPARs are related to retinoid, glucocorticoid, and thyroid hormone receptors [3]. They regulate diverse physiological processes, such as lipid biosynthesis and glucose metabolism, by binding to sequence-specific PPAR response elements in the promoter region of target genes. Recently, PPARs and their ligands have been found to inhibit the expression of proinflammatory genes, implicating them as regulators of immune and inflammatory responses. Several studies have demonstrated that PPAR ligands possess anti-inflammatory properties that may prove useful in the treatment of inflammatory lung diseases [4–6].

PPAR*γ* is the most extensively studied PPAR subtype. It is involved in a series of lung diseases, including lung fibrosis, pulmonary vascular diseases, acute lung injury, and lung cancer [7–10]. Besides its regulatory role in adipocyte differentiation, glucose, and lipid metabolism, PPAR*γ* activation reduces the synthesis and release of immunomodulatory cytokines from various cell types that participate in the regulation of inflammatory and immune processes. Specifically, mounting evidence suggests that PPAR*γ* plays important roles in regulating processes related to airway inflammation, airway remodeling, and airway hyperresponsiveness, indicating that PPAR*γ* and its ligand show potential as targets to develop treatments for chronic airway inflammatory diseases [11]. In fact, PPAR*γ* has been implicated in AMH. Recent studies have shown that chronic airway inflammatory diseases in humans are associated with altered PPAR**γ** expression and that PPAR**γ** ligand can attenuate AMH in both* in vitro* and *in vivo *experimental models. This paper will summarize recent work implicating PPAR**γ** in AMH.

## 2. AMH and Chronic Airway Inflammatory Diseases

### 2.1. Significance of AMH

Mucus secretion is essential for protecting airways. It is vital for air humidification, warming, and cleaning [12]. However, in patients with chronic airway inflammatory diseases, excessive mucus is secreted into the airway, which leads to hospitalization and death in many patients. This hypersecretion, termed AMH, obstructs the airways, limits airflow, impairs gas change, and causes ventilation-perfusion mismatch. Patients with chronic airway inflammatory diseases that also present with AMH generally have poor lung function and high rates of hospitalization and death. Compromised mucociliary function can reduce mucus clearance, which can encourage bacterial colonization, leading to chronic pulmonary infections and exacerbations. In addition, chemicals synthesized by bacteria or released when bacteria are degraded by the immune system can stimulate mucin synthesis and mucus secretion, resulting in a vicious circle [1, 13]. 

Studies suggest that AMH is not merely a clinical symptom but instead a critical factor in the pathogenesis of chronic airway inflammatory diseases. AMH is associated with greater susceptibility to COPD, a decline in forced expiratory volume in one second, hospitalization, and excess mortality [14–16]. The incidence of COPD in young adults presenting with AMH symptoms is three-fold higher than that in subjects who have never reported AMH symptoms, regardless of their smoking habits [17]. These findings are supported by an analysis of the Framingham offspring cohort studies [18]. 

AMH has long been recognized as a major cause of death in asthma. One study found luminal occlusions covering 20–100% of the cross-sectional area in patients who died from asthma, leading the authors to conclude that luminal obstruction of airways by an exudate composed of mucus and cells is a major contributor to asthma fatality [19]. In addition, AMH symptoms such as chronic cough or phlegm are independently and significantly related to uncontrolled asthma, and a history of persistent symptoms related to AMH is associated with a more severe asthma phenotype [20]. AMH also contributes to morbidity due to cystic fibrosis (CF). In CF patients, the reduced ability of epithelial cells to secrete chloride usually leads to reduced mucus accumulation in the airways, which favors chronic infection by *Pseudomonas aeruginosa *and other organisms. AMH contributes to CF morbidity by increasing the frequency and severity of pulmonary infections as well as by impairing lung function [21]. 

### 2.2. Inflammation and AMH

Abnormal inflammatory response is the major component of chronic airway inflammatory diseases. Such inflammatory response has been associated with AMH in airway diseases. Numerous studies have demonstrated that inflammatory stimuli and mediators/cytokines contribute to excess mucin synthesis and mucus secretion in airways. For instance, cigarette smoke, neutrophil elastase, and *P.  aeruginosa* proteases are well-known inflammatory stimuli that can cause significant inflammatory responses, which result in goblet cell metaplasia and hyperplasia, leading in turn to mucin overproduction and mucus hypersecretion in airways [22–24]. Inflammatory cytokines such as tumor necrosis factor-*α* (TNF-*α*), interleukin (IL)-1*β*, IL-6, IL-8, IL-13, and IL-17 can upregulate the expression of MUC5AC, the marker of goblet cell metaplasia, through different signaling pathways [25–29]. This is the most important event in the pathogenesis of AMH. Thus, the ability to control airway inflammatory responses should reduce AMH and thereby benefit patients with chronic airway inflammatory diseases. However, current anti-inflammatory treatments with corticosteroids are not effective in all patients with chronic airway inflammatory diseases. Thus, the search for novel drug targets for these diseases continues.

In summary, AMH arising as a result of inflammatory processes can lead to physiologically and clinically measurable mechanical airway obstruction in patients with chronic airway inflammatory diseases. In this way, AMH significantly affects the pathogenesis, progression, and prognosis of these diseases. Therapies that aim to control the inflammation that triggers AMH may be effective at treating chronic airway inflammatory diseases [30–32].

## 3. Expression and Anti-Inflammatory Role of PPAR*γ* in the Lung

### 3.1. Expression of PPAR*γ* in Lung Cells

PPAR**γ** is expressed in many types of structural cells in the lung, including fibroblasts, ciliated airway epithelial cells, alveolar type II pneumocytes, and airway smooth muscle cells [3, 4, 32]. A study in PPAR**γ*-*knockout mice has highlighted the role of this transcription factor in animal models. A specific PPAR**γ** deletion in airway epithelium produced persistent enlargement of the airspaces in adult mice, which showed more severe emphysema symptoms and higher macrophage numbers after exposure to cigarette smoke than did mice carrying the PPAR*γ*  gene [33]. These results suggest that epithelial PPAR**γ** is required for proper lung maturation and response to injury. 

PPAR**γ** is also present in several types of inflammatory, immune cells in the lung, where it exerts anti-inflammatory and immuno-modulatory effects. Specially, PPAR**γ** has been found in macrophages, eosinophils, T cells, B cells, and dendritic cells [34–38]. It plays an anti-inflammatory role in lung cells mainly by inhibiting cytokine production and enhancing phagocytosis of apoptotic neutrophils, a process essential to resolve inflammation. 

Increasing evidence suggests that altered PPAR**γ** expression and function may be associated with chronic airway inflammatory diseases and that stimulation of this receptor may attenuate inflammatory responses. A study examining the expression of PPAR*γ* in lung tissues of patients with COPD found the protein to be present mainly in alveolar epithelial cells and smooth muscle cells in the bronchial walls. The intensity of PPAR*γ* was inversely proportional to the severity of the COPD, with the signal stronger in patients with milder COPD [39]. Another study found that PPAR*γ* is expressed in CF- and non-CF-type human airway epithelial cells, but appears to be either less abundant, less functional in binding its target DNA sequence, or both, in CF [40]. In asthmatic individuals, but not healthy objects, segmental allergen challenge led to downregulation of PPAR*γ* mRNA and protein expression in alveolar macrophages [41]. These findings are suggestive of a role for PPAR*γ* agonists in the treatment of chronic lung inflammatory diseases.

### 3.2. The Anti-Inflammatory Role of PPAR*γ*

Building on the evidence linking PPAR*γ* and airway inflammatory diseases, several studies have investigated the therapeutic role of the receptor and its ligand in cellular and animal models of these diseases. Administration of PPAR*γ* ligand to BALB/c mice sensitized and challenged with ovalbumin (OVA) reduced the levels of proinflammatory mediators in bronchoalveolar lavage fluid (BALF) and attenuated the inflammatory response in the lung [35, 42, 43]. Treating human airway epithelial cell line A549 with the PPAR*γ* agonist troglitazone blocked the ability of phorbol 12-myristate 13-acetate to stimulate an increase in TNF-*α* levels. However, this effect of troglitazone required the presence of MUC1/Muc1 [44]. The authors concluded that PPAR*γ* exerts an anti-inflammatory effect by stimulating MUC1/Muc1 expression, which then blocks the production of TNF-*α*/IL-8 induced by phorbol 12-myristate 13-acetate in airway epithelial cells [44]. In addition, PPAR*γ* plays an essential role in the pathway through which monocyte/macrophage-derived microparticles activate NF-*κ*B and ultimately induce upregulation of proinflammatory mediators in human lung epithelial cells [45].

The anti-inflammatory effect of PPAR*γ* is thought to be due mainly to its ability to downregulate proinflammatory gene expression. It does so by sequestering shared coactivators, reducing the ability of inflammatory transcription factors to bind their target DNA. The second way is through ligand-dependent transrepression, preventing other transcription factors' association with DNA sequences. At the same time, PPAR*γ* can increase the transcription of anti-inflammatory genes. Agonist binding to PPAR*γ* induces a conformational change in the receptor, allowing the dissociation of corepressors and the association of coactivator molecules. This allows the formation of PPAR*γ* dimmers or heterodimers and subsequent interaction with peroxisome proliferator response elements, which increases anti-inflammatory gene transcription [46].

Taken together, these findings suggest that PPAR*γ* participates in both physiological and pathological processes in chronic airway inflammatory diseases. These studies make a strong case for PPAR*γ* activation as a potential treatment for chronic airway inflammatory diseases. 

## 4. PPAR*γ* and AMH

Since the inflammatory response is the major contributor to AMH, and PPAR**γ** and its ligand help regulate airway inflammation, several studies have investigated the potential involvement of PPAR**γ** and its ligand in AMH.

Lee and colleagues carried out the first study to examine the role of PPAR**γ** ligand on mucin production in human airway epithelial NCI-H292 cells exposed to cigarette smoke [47]. Exposure caused a significant increase in TNF-*α* and MUC5AC production, which was attenuated by administration of the PPAR*γ* agonist rosiglitazone. Rosiglitazone increased the expression of phosphatase and tensin homolog deleted on chromosome 10 (PTEN) and inhibited the PI3K/Akt pathway. PTEN antagonizes PI3K-mediated signaling, whose role in mucin production is well documented [47, 48]. These results provide the first evidence that a treatment targeting PPAR*γ* can significantly inhibit cigarette smoke-induced mucin production, raising the possibility that this approach may be useful in the clinic. Indeed, the study authors proposed that using a PPAR*γ* agonist to activate PTEN and inhibit PI3K/Akt may be a potential therapy for cigarette smoke-induced mucin secretory diseases [47].

Subsequent studies evaluated the effect of rosiglitazone on airway mucin production in animal models of AMH. Exposing rats for two weeks to acrolein, one of the most toxic components in cigarette smoke, induced goblet cell hyperplasia in bronchial epithelium and upregulated MUC5AC mRNA and protein expression in rat lungs. These changes were associated with airway inflammation, as evidenced by the increased numbers of inflammatory cells and levels of inflammatory cytokines (IL-1*β*, IL-8, and TNF-*α*) in BALF. Treating the rats with rosiglitazone before acrolein exposure attenuated these changes in a dose-dependent manner [49]. Rosiglitazone has also been shown to inhibit NF-*κ*B activation, which is well known to play an important role in regulating not only multiple inflammatory cytokines but also AMH in inflammatory lung diseases [50, 51]. Therefore, inhibition of NF-*κ*B activation by rosiglitazone may explain the observed downregulation of proinflammatory mediators and the reduction in AMH. From these findings, Liu et al. concluded that ligand-induced activation of PPAR*γ* may be a novel therapy for AMH in chronic airway inflammatory diseases such as COPD [49].

AMH is also an important pathologic feature of asthma, which is characterized by nonspecific airway inflammation. Several studies have examined the involvement of PPAR*γ* and its ligand on AMH in a mouse model of asthma. AMH was observed in airways of animals sensitized and challenged with OVA, while it was barely detectable in airways of nonsensitized animals. Administration of the PPAR*γ* agonist ciglitazone via nebulizer reduced OVA-induced mucus gland hyperplasia and airway occlusion due to mucus hypersecretion by approximately 75% [52]. In a different mouse model, mice were exposed or not to toluene diisocyanate to induce asthma. Exposed mice that received PPAR*γ* agonists (rosiglitazone or pioglitazone) or adenovirus carrying PPAR*γ*2 cDNA showed a lower percentage of airway epithelium staining positive with PAS than did exposed mice that did not receive agonists or adenovirus [53]. In addition, both of these studies found that PPAR*γ* agonists support protective remodeling of the airway wall by regulating TGF-*β* or TGF-*β*1 and collagen deposition. Results from both of these mouse models support the possibility of using PPAR*γ* agonist to treat AMH in humans.

In addition to these observations in cell lines or animal models, several lines of indirect evidence link activation of PPAR*γ* and AMH. Activation of PPAR*γ* by rosiglitazone or pioglitazone markedly reduced mRNA expression of matrix metalloproteinase-9 (MMP-9) and inhibited TNF-*α*  induced MMP-9 gelatinolytic activity [54]. Since MMP-9 is an important MMP related to AMH [55], these results suggest that PPAR*γ* may attenuate AMH through a pathway involving MMP-9. Simvastatin is a statin that modulates inflammatory processes and that can inhibit mucin production and AMH [56]. A recent study indicated that its anti-inflammatory effect may involve PPAR*γ* activation [57], which means that simvastatin may suppress AMH via a mechanism mediated by PPAR*γ*. It seems clear from these diverse lines of evidence that the effects of PPAR*γ* and its ligand on AMH are mediated by multiple mechanisms. Further work is needed to identify new ligands of PPAR*γ* and investigate the signaling pathways affected by the receptor. 

In summary, substantial evidences indicate that PPAR*γ* is involved in AMH and that PPAR*γ* ligand suppresses AMH by inhibiting proinflammatory cytokines and inflammatory signaling pathways. Therefore, PPAR*γ* agonists may have potential as treatments for AMH in chronic airway inflammatory diseases.

## 5. Considerations for Targeting PPAR*γ* in the Treatment of AMH

Although several studies have suggested that PPAR*γ* is involved in AMH and that PPAR*γ* agonists inhibit AMH, there is still a long road ahead before laboratory studies can be translated to the clinic. For one thing, the pathogenesis of chronic airway inflammatory diseases such as COPD, asthma, and CF is extremely complicated. Only a few studies, most of them descriptive, have directly investigated the role of PPAR*γ* and PPAR*γ* ligand in AMH. As a result, the signaling pathways affected by PPAR*γ* or PPAR*γ* agonists remain poorly understood. Further investigation of these areas will be required to define the effects of PPAR*γ* ligands on airway epithelial cells and the mechanisms by which these pro- and/or anti-inflammatory responses occur. 

A second consideration for targeting PPAR*γ* in the clinic is that the route of PPAR*γ* agonist administration may be important. In Honda's study [52], treating Balb/c mice with nebulized ciglitazone led to a reduction in mucus production in the airways, whereas orally administered ciglitazone had no such effect [58]. Oral gavage of rosiglitazone or pioglitazone reduced toluene diisocyanate-induced AMH in Balb/c mice [53], while oral rosiglitazone treatment had no effect on goblet cell hyperplasia in C57BL/6 mice [59]. Future work should clarify whether the different results observed with different agonists or models of administration reflect differences in the sensitivity of mouse strains to regulation of airway inflammation and AMH, or whether agonist identity and mode of administration are crucial.

Finally, the safety of PPAR*γ* agonists in patients with chronic airway inflammatory disease should be carefully examined. Recent studies suggest an association between rosiglitazone treatment and increased risk of cardiovascular events in patients with type 2 diabetes [60, 61]. Thus it remains to be seen whether PPAR*γ* agonist is safe for patients with chronic airway inflammatory diseases. 

## 6. Summary

The present paper describes the clinical importance of AMH in the pathogenesis of chronic airway inflammatory diseases and makes the case for exploring PPAR*γ* and PPAR*γ* ligand as potential target in treating AMH. Corticosteroid is the first-line anti-inflammatory drug used to chronic airway inflammatory diseases, but its therapeutic effects are controversial, and physicians have begun to pay attention to its undesirable side effects [62]. In fact, it is generally accepted that steroids have only limited effects on AMH [63], so there is a demand for novel and effective anti-inflammatory drugs that can reduce AMH. Increasing evidence suggests that PPAR*γ* is involved in mucin production and that PPAR*γ* agonists inhibit AMH both *in vitro* and *in vivo*. Indeed, a recent clinical trial found that rosiglitazone improved lung function in steroid-resistant asthma patients [64], providing direct evidence that patients with chronic airway inflammatory diseases can benefit from PPAR*γ* agonist treatment. Thus, PPAR*γ* agonists may represent a novel class of pharmacological agents useful in the management of AMH in chronic airway inflammatory diseases. 

## References

[1] Fahy JV, Dickey BF (2010). Medical progress: airway mucus function and dysfunction. *New England Journal of Medicine*.

[2] Wen FQ, Shen YC (2011). Expectorant therapy revisited in chronic obstructive pulmonary disease. *Chinese Journal of Tuberculosis and Respiratory Diseases*.

[3] Berger J, Moller DE (2002). The mechanisms of action of PPARs. *Annual Review of Medicine*.

[4] Belvisi MG, Mitchell JA (2009). Targeting PPAR receptors in the airway for the treatment of inflammatory lung disease. *British Journal of Pharmacology*.

[5] Ward JE, Tan X (2007). Peroxisome proliferator activated receptor ligands as regulators of airway inflammation and remodelling in chronic lung disease. *PPAR Research*.

[6] Remels AH, Gosker HR, Schrauwen P, Langen RC, Schols AM (2008). Peroxisome proliferator-activated receptors: a therapeutic target in COPD?. *European Respiratory Journal*.

[7] Lakatos HF, Thatcher TH, Kottmann RM, Garcia TM, Phipps RP, Sime PJ (2007). The role of PPARs in lung fibrosis. *PPAR Research*.

[8] Nisbet RE, Sutliff RL, Hart CM (2007). The role of peroxisome proliferator-activated receptors in pulmonary vascular disease. *PPAR Research*.

[9] Di Paola R, Cuzzocrea S (2007). Peroxisome proliferator-activated receptors and acute lung injury. *PPAR Research*.

[10] Keshamouni VG, Han S, Roman J (2007). Peroxisome proliferator-activated receptors in lung cancer. *PPAR Research*.

[11] Belvisi MG, Hele DJ, Birrell MA (2006). Peroxisome proliferator-activated receptor gamma agonists as therapy for chronic airway inflammation. *European Journal of Pharmacology*.

[12] Rogers DF (1994). Airway goblet cells: responsive and adaptable frontline defenders. *European Respiratory Journal*.

[13] Sethi S, Murphy TF (2008). Infection in the pathogenesis and course of chronic obstructive pulmonary disease. *New England Journal of Medicine*.

[14] Speizer FE, Fay ME, Dockery DW, Ferris BG (1989). Chronic obstructive pulmonary disease mortality in six U.S. cities. *American Review of Respiratory Disease*.

[15] Vestbo J, Prescott E, Lange P (1996). Association of chronic mucus hypersecretion with FEV1 decline and chronic obstructive pulmonary disease morbidity. *American Journal of Respiratory and Critical Care Medicine*.

[16] Burgel PR, Nesme-Meyer P, Chanez P (2009). Cough and sputum production are associated with frequent exacerbations and hospitalizations in COPD subjects. *Chest*.

[17] De Marco R, Accordini S, Cerveri I (2007). Incidence of chronic obstructive pulmonary disease in a cohort of young adults according to the presence of chronic cough and phlegm. *American Journal of Respiratory and Critical Care Medicine*.

[18] Kohansal R, Martinez-Camblor P, Agustí A, Sonia Buist A, Mannino DM, Soriano JB (2009). The natural history of chronic airflow obstruction revisited: an analysis of the Framingham Offspring Cohort. *American Journal of Respiratory and Critical Care Medicine*.

[19] Kuyper LM, Paré PD, Hogg JC (2003). Characterization of airway plugging in fatal asthma. *American Journal of Medicine*.

[20] Siroux V, Boudier A, Bousquet J (2009). Phenotypic determinants of uncontrolled asthma. *Journal of Allergy and Clinical Immunology*.

[21] Henke MO, Ratjen F (2007). Mucolytics in cystic fibrosis. *Paediatric Respiratory Reviews*.

[22] Baginski TK, Dabbagh K, Satjawatcharaphong C, Swinney DC (2006). Cigarette smoke synergistically enhances respiratory mucin induction by proinflammatory stimuli. *American Journal of Respiratory Cell and Molecular Biology*.

[23] Arai N, Kondo M, Izumo T, Tamaoki J, Nagai A (2010). Inhibition of neutrophil elastase-induced goblet cell metaplasia by tiotropium in mice. *European Respiratory Journal*.

[24] Klinger JD, Tandler B, Liedtke CM, Boat TF (1984). Proteinases of Pseudomonas aeruginosa evoke mucin release by tracheal epithelium. *Journal of Clinical Investigation*.

[25] Lora JM, Zhang DM, Liao SM (2005). Tumor necrosis factor-*α* triggers mucus production in airway epithelium through an I*κ*B kinase *β*-dependent mechanism. *Journal of Biological Chemistry*.

[26] Fujisawa T, Velichko S, Thai P, Hung LY, Huang F, Wu R (2009). Regulation of airway MUC5AC expression by IL-1*β* and IL-17A; the NF-*κ*B paradigm. *Journal of Immunology*.

[27] Chen Y, Thai P, Zhao YH, Ho YS, DeSouza MM, Wu R (2003). Stimulation of airway mucin gene expression by interleukin (IL)-17 through IL-6 paracrine/autocrine loop. *Journal of Biological Chemistry*.

[28] Bautista MV, Chen Y, Ivanova VS, Rahimi MK, Watson AM, Rose MC (2009). IL-8 regulates mucin gene expression at the posttranscriptional level in lung epithelial cells. *Journal of Immunology*.

[29] Zhert G, Sung WP, Nguyenvu LT (2007). IL-13 and epidermal growth factor receptor have critical but distinct roles in epithelial cell mucin production. *American Journal of Respiratory Cell and Molecular Biology*.

[30] Evans CM, Koo JS (2009). Airway mucus: the good, the bad, the sticky. *Pharmacology and Therapeutics*.

[31] Decramer M, Janssens W (2010). Mucoactive therapy in copd. *European Respiratory Review*.

[32] Belvisi MG, Hele DJ (2008). Peroxisome proliferator-activated receptors as novel targets in lung disease. *Chest*.

[33] Simon DM, Arikan MC, Srisuma S (2006). Epithelial cell PPAR*γ* is an endogenous regulator of normal lung maturation and maintenance. *Proceedings of the American Thoracic Society*.

[34] Asada K, Sasaki S, Suda T, Chida K, Nakamura H (2004). Antiinflammatory roles of peroxisome proliferator-activated receptor *γ* in human alveolar macrophages. *American Journal of Respiratory and Critical Care Medicine*.

[35] Woerly G, Honda K, Loyens M (2003). Peroxisome proliferator-activated receptors *α* and *γ* down-regulate allergic inflammation and eosinophil activation. *Journal of Experimental Medicine*.

[36] Yang XY, Wang LH, Chen T (2000). Activation of human T lymphocytes is inhibited by peroxisome proliferator-activated receptor *γ* (PPAR*γ*) agonists. PPAR*γ* co-association with transcription factor NFAT. *Journal of Biological Chemistry*.

[37] Chinetti G, Griglio S, Antonucci M (1998). Activation of proliferator-activated receptors *α* and *γ* induces apoptosis of human monocyte-derived macrophages. *Journal of Biological Chemistry*.

[38] Faveeuw C, Fougeray S, Angeli V (2000). Peroxisome proliferator-activated receptor *γ* activators inhibit interleukin-12 production in murine dendritic cells. *FEBS Letters*.

[39] Li J, Dai A, Hu R, Zhu L, Tan S (2010). Positive correlation between PPAR*γ*/PGC-1*α* and *γ*-GCS in lungs of rats and patients with chronic obstructive pulmonary disease. *Acta Biochimica et Biophysica Sinica*.

[40] Perez A, Van Heeckeren AM, Nichols D, Gupta S, Eastman JF, Davis PB (2008). Peroxisome proliferator-activated receptor-*γ* in cystic fibrosis lung epithelium. *American Journal of Physiology*.

[41] Kobayashi M, Thomassen MJ, Rambasek T (2005). An inverse relationship between peroxisome proliferator-activated receptor *γ* and allergic airway inflammation in an allergen challenge model. *Annals of Allergy, Asthma and Immunology*.

[42] Trifilieff A, Bench A, Hanley M, Bayley D, Campbell E, Whittaker P (2003). PPAR-*α* and -*γ* but not -*δ* agonists inhibit airway inflammation in a murine model of asthma: in vitro evidence for an NF-*κ*B-independent effect. *British Journal of Pharmacology*.

[43] Lee KS, Park SJ, Hwang PH (2005). PPAR-gamma modulates allergic inflammation through up-regulation of PTEN. *FASEB Journal*.

[44] Park YS, Lillehoj EP, Kato K, Park CS, Kim KC (2012). PPAR*γ* inhibits airway epithelial cell inflammatory response through a MUC1-dependent mechanism. *American Journal of Physiology*.

[45] Neri T, Armani C, Pegoli A (2011). Role of NF-*κ*B and PPAR-*γ* in lung inflammation induced by monocyte-derived microparticles. *European Respiratory Journal*.

[46] Straus DS, Glass CK (2007). Anti-inflammatory actions of PPAR ligands: new insights on cellular and molecular mechanisms. *Trends in Immunology*.

[47] Lee SY, Kang EJ, Hur GY (2006). Peroxisome proliferator-activated receptor-*γ* inhibits cigarette smoke solution-induced mucin production in human airway epithelial (NCI-H292) cells. *American Journal of Physiology*.

[48] Wu Q, Jiang D, Chu HW Cigarette smoke induces growth differentiation factor 15 production in human lung epithelial cells: implication in mucin over-expression.

[49] Liu DS, Liu WJ, Chen L (2009). Rosiglitazone, a peroxisome proliferator-activated receptor-*γ* agonist, attenuates acrolein-induced airway mucus hypersecretion in rats. *Toxicology*.

[50] Imanifooladi AA, Yazdani S, Nourani MR (2010). The role of nuclear factor-*κ*B in inflammatory lung disease. *Inflammation and Allergy*.

[51] Ou XM, Feng YL, Wen FQ (2008). Macrolides attenuate mucus hypersecretion in rat airways through inactivation of NF-*κ*B. *Respirology*.

[52] Honda K, Marquillies P, Capron M, Dombrowicz D (2004). Peroxisome proliferator-activated receptor *γ* is expressed in airways and inhibits features of airway remodeling in a mouse asthma model. *Journal of Allergy and Clinical Immunology*.

[53] Lee KS, Park SJ, Kim SR (2006). Modulation of airway remodeling and airway inflammation by peroxisome proliferator-activated receptor *γ* in a murine model of toluene diisocyanate-induced asthma. *Journal of Immunology*.

[54] Hetzel M, Walcher D, Grüb M, Bach H, Hombach V, Marx N (2003). Inhibition of MMP-9 expression by PPAR*γ* activators in human bronchial epithelial cells. *Thorax*.

[55] Ren S, Guo LL, Yang J (2011). Doxycycline attenuates acrolein-induced mucin production, in part by inhibiting MMP-9. *European Journal of Pharmacology*.

[56] Chen YJ, Chen P, Wang HX (2010). Simvastatin attenuates acrolein-induced mucin production in rats: involvement of the Ras/extracellular signal-regulated kinase pathway. *International Immunopharmacology*.

[57] Shen Y, Wu H, Wang C (2010). Simvastatin attenuates cardiopulmonary bypass-induced myocardial inflammatory injury in rats by activating peroxisome proliferator-activated receptor *γ*. *European Journal of Pharmacology*.

[58] Mueller C, Weaver V, Vanden Heuvel JP, August A, Cantorna MT (2003). Peroxisome proliferator-activated receptor *γ* ligands attenuate immunological symptoms of experimental allergic asthma. *Archives of Biochemistry and Biophysics*.

[59] Ward JE, Fernandes DJ, Taylor CC, Bonacci JV, Quan L, Stewart AG (2006). The PPAR*γ* ligand, rosiglitazone, reduces airways hyperresponsiveness in a murine model of allergen-induced inflammation. *Pulmonary Pharmacology and Therapeutics*.

[60] Nissen SE, Wolski K (2007). Effect of rosiglitazone on the risk of myocardial infarction and death from cardiovascular causes. *New England Journal of Medicine*.

[61] Dahabreh IJ (2008). Meta-analysis of rare events: an update and sensitivity analysis of cardiovascular events in randomized trials of rosiglitazone. *Clinical Trials*.

[62] Drummond MB, Dasenbrook EC, Pitz MW, Murphy DJ, Fan E (2008). Inhaled corticosteroids in patients with stable chronic obstructive pulmonary disease: a systematic review and meta-analysis. *Journal of the American Medical Association*.

[63] Balsamo R, Lanata L, Egan CG (2010). Mucoactive drugs. *European Respiratory Review*.

[64] Spears M, Donnelly I, Jolly L (2009). Bronchodilatory effect of the PPAR-*γ* agonist rosiglitazone in smokers with asthma. *Clinical Pharmacology and Therapeutics*.

